# The prognostic value of positive T-wave in lead aVR in hemodialysis patients

**DOI:** 10.1007/s10157-015-1100-8

**Published:** 2015-02-28

**Authors:** Andrzej Jaroszyński, Anna Jaroszyńska, Janusz Siebert, Wojciech Dąbrowski, Jarosław Niedziałek, Anna Bednarek-Skublewska, Tomasz Zapolski, Andrzej Wysokiński, Wojciech Załuska, Andrzej Książek, Todd T. Schlegel

**Affiliations:** 1 Department of Family Medicine, Medical University of Lublin, Staszica 11, 20-081 Lublin, Poland; 2 Department of Cardiology, Medical University of Lublin, Lublin, Poland; 3 Department of Family Medicine, University Center for Cardiology, Medical University of Gdańsk, Gdańsk, Poland; 4 Department of Anesthesiology and Intensive Care, Medical University of Lublin, Lublin, Poland; 5 Department of Nephrology, Medical University of Lublin, Lublin, Poland; 6 NASA Johnson Space Center, Houston, TX USA

**Keywords:** Electrocardiogram, End-stage renal disease, T-wave amplitude, Lead aVR, Cardiovascular mortality, Sudden cardiac death

## Abstract

**Background:**

Given that cardiac disease is the leading cause of mortality in hemodialysis (HD) patients, identification of patients at risk for cardiac mortality is crucial. The aim of this study was to determine if positive T-wave amplitude in lead aVR (TaVR) was predictive of cardiovascular (CV) mortality and sudden cardiac death (SCD) in a group of HD patients.

**Methods and results:**

After exclusion, 223 HD patients were prospectively followed-up for 25.43 ± 3.56 months. Patients were divided into TaVR negative (*n* = 186) and TaVR positive (*n* = 37) groups. Myocardial infarction, diabetes and beta-blocker therapy were more frequent in positive TaVR patients. Patients with upright TaVR were older, had higher left ventricular mass index, lower ejection fraction, higher calcium × phosphate product, higher troponin T level, higher prevalence of ST-T abnormalities, and increased width of QRS complex and QT interval, compared with patients with negative TaVR. A Kaplan–Meier analysis showed that the cumulative incidences of CV mortality as well as SCD were higher in patients with positive TaVR compared with those with negative TaVR (log-rank, *p* < 0.001 in both cases). A multivariate analysis selected age [hazard ratio (HR) 1.71, *p* < 0.001], heart rate (HR 1.42, *p* = 0.016), and positive TaVR (HR 2.21, *p* = 0.001) as well as age (HR 1.88, *p* < 0.001), and positive TaVR (HR 1.53, *p* = 0.014) as independent predictors of CV mortality and SCD, respectively.

**Conclusion:**

In HD patients, positive TaVR is an independent and powerful predictor of CV mortality as well as SCD. This simple ECG parameter provides additional information beyond what is available with other known traditional risk factors and allows the identification of patients most at risk of CV events.

## Introduction

Cardiovascular (CV) diseases are the leading cause of mortality in hemodialysis (HD) patients, accounting for nearly half of all deaths in this patient population. The CV mortality rate of HD patients is higher by a factor of ~10 than that of the general population, even after adjustment for age, race, gender, and comorbidities [1–4]. In particular, SCD has been identified as an important contributor of mortality. SCD is the single largest cause of death in HD patients responsible for 22–29 % of all-cause mortality [1–3, 5]. Given the extremely high incidence of CV diseases as well as the case fatality rate of cardiac events, early identification of individuals at risk for increased CV mortality is clinically highly relevant.

In cardiac diseases ECG is beneficial not only as a diagnostic but also prognostic tool. Various ECG criteria and scores have demonstrated association with CV. T-wave abnormalities are among the most frequently encountered pathologic ECG findings in apparently healthy populations. The prognostic significance of T-wave abnormalities has been extensively investigated. For example, the presence of even isolated minor T-wave abnormalities is associated with increased long-term cardiac mortality [6–9].

ECG abnormalities are very common in HD patients, with ST-T changes observed in 40–60 %. Moreover, volume and electrolyte status can influence various ECG variables, with ST-T changes frequently appearing in relation to HD timing and the HD process itself often inducing or potentiating ECG alterations, which additionally might compromise ECG interpretation [9, 10].

The prognostic role of T-wave changes in lead aVR has long been underestimated. Several recent studies have validated the role of upright T-wave amplitude in lead aVR (TaVR) as a powerful, independent prognostic predictor of CV mortality in the general population [11–13] as well as in some clinical settings [14–17]. However, neither the prevalence nor the predictive value of a positive TaVR has been evaluated in HD patients.

We designed this study to prospectively determine whether TaVR was predictive of CV mortality and SCD in a group of HD patients.

## Materials and methods

### Patients

The study included adult chronic HD patients treated at dialysis centers in Lublin. The exclusion criteria were: HD treatment less than 1 month, cardiac pacing, preexcitation, atrial fibrillation, right or left bundle branch block, and noncardiac diseases limiting the chance of a 1-year survival. All patients gave a written consent, and the studies were approved by members of the local ethics committees.

### Electrocardiography

**Fig. 1:**
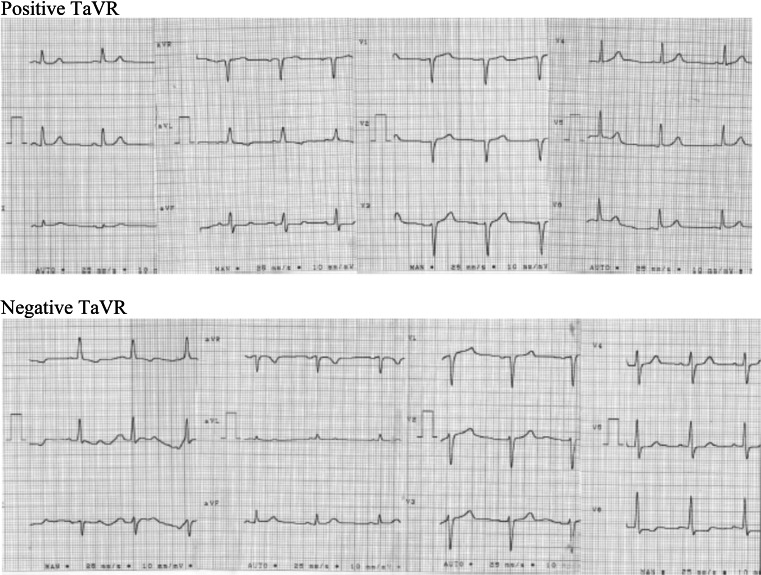
T-wave morphology in lead aVR. *Positive* TaVR was defined as a wave with a *positive* deflection >0 mV. *Negative* TaVR was defined as TaVR ≤ 0 mm

Surface 12-lead resting ECGs were recorded with a computer-based electrocardiograph (Cardioperfect, version 1.1, CardioControl NV, Rijswijk, The Netherlands). ECGs were conducted the day after a dialysis session while subjects were lying in the supine position and breathing normally after at least 5 min of rest. Values of TaVRs were recorded automatically by the ECG machine. Positive TaVR was defined as a wave with a positive deflection >0 mV. Negative TaVR was defined as TaVR ≤ 0 mm. The representative ECGs of positive and negative TaVR are presented in Fig. 1. The width of QRS complex and QT interval were recorded automatically by the ECG machine. ST depression was defined as more than 0.5 drop in ST-segment in lead V5 from the isoelectric line [11]. ST elevation in lead aVR was defined as the elevation ≥8 μV from the isoelectric line [17].

### Biochemical measurements

The following parameters were measured by automated analyzers at the beginning of the evaluation: serum sodium, potassium, calcium, phosphate, creatinine, urea, haemoglobin, intact parathormone (PTH), albumin, C-reactive protein (CRP), total cholesterol, high-density lipoprotein (HDL) cholesterol and triglycerides (TG). Low-density lipoprotein (LDL) cholesterol was calculated using the Friedewald equation: LDL (mg/dL) = total cholesterol − HDL − (triglycerides/5). Cardiac TnT in plasma was measured before dialysis by electrochemiluminescence immunoassay (Elecsys 2010 analyser, Roche Diagnostics) with a detection limit of 0.01 μg/L. Blood was obtained after at least 8 h fasting.

### Echocardiographic examination

Two-dimensional echocardiographic examination was performed using a 2.5-MHz transducer by the cardiologist who was blinded to the clinical data of the study subjects. The LVM was calculated according to the formula of Devereux and Reicheck [18] and this was indexed for body surface to obtain the LVMI. Left ventricular hypertrophy (LVH) was defined by an LVMI over 131 g/m^2^ in males or over 110 g/m^2^ in females [19]. All echocardiographic measurements were performed in the morning after dialysis [19] according to the recommendations of the American Society of Echocardiography.

### Follow-up data

All patients were followed for at least 3 years after the day of the baseline assessment or until death or renal transplant. CV mortality and SCD were used as endpoints. CV death was defined according to Standardized Definitions for End Point Events in Cardiovascular Trials [20]. SCD was defined according to Hemodialysis (HEMO) trial as witnessed and unwitnessed unexpected deaths, with a preceding duration of symptoms less than 24 h for witnessed deaths, and less than the interval since the last dialysis session for unwitnessed deaths [2]. All events were independently classified by two physicians. In the case of disagreement, the event was verified by an expert in cardiology.

### Statistical analysis

Statistical analysis was carried out on an IBM PC using Statistica Version 10. Results were tested for normality by using Kolmogorov–Smirnov test. When normally distributed, continuous variables were expressed as mean ± SD, and as median and range when non-normally distributed. Continuous data were compared using the Student *t*-test when normally distributed and using Mann–Whitney *U*-test when non-normally distributed. Categorical data were expressed as frequencies and percentages and were compared using the *χ* ^2^ test. Linear regression analysis was assessed using the Pearson correlation coefficient or Spearmans’ rank correlation coefficient when appropriate. For the survival analysis, patients were divided into TaVR negative and TaVR positive groups. Survival was measured beginning from the day of baseline examination until death or censoring. If patients underwent renal transplantation they were censored on the day of their last dialysis. Cumulative survival curves were constructed by using the Kaplan–Meier method for CV mortality and SCD. Differences between patient groups were assessed by use of the log-rank test. Relationships between baseline parameters and endpoints were analyzed with Cox proportional hazard regression analysis. In the univariate analyses, parameters that showed differences between TaVR positive and negative groups were entered. Explanatory variables with a *p* value < 0.01 in the univariate analysis were entered into a multivariate analysis. Probability values of *p* < 0.05 were accepted as significant.

## Results

Of the total of 321 HD patients initially identified, 98 patients were excluded due to atrial fibrillation, noncardiac diseases limiting the chance of a 1-year survival, pacemaker rhythm, and bundle branch blocks. The remaining 223 HD patients (115 females and 108 males), aged 47–82 years (mean 67.67 ± 6.96), who remained on HD from 2 to 94 months (mean 40.01 ± 20.34) entered the study. The causes of end-stage renal disease (ESRD) were diabetes (*n* = 91), chronic glomerulonephritis (*n* = 46), hypertensive nephropathy (*n* = 22), obstructive nephropathy (*n* = 9), polycystic kidney disease (*n* = 9), chronic pyelonephritis (*n* = 6), and unknown (*n* = 40). None of the HD patients received anti-arrhythmics (Class I or III) or digitalis. Out of 223 patients who qualified for the study, 168 (75.3 %) were taking angiotensin-converting enzyme inhibitors/angiotensin receptor blockers, 159 (71.3 %) beta-blockers, 81 (36.3 %) statins and 118 (52.9 %) calcium blockers.

Positive TaVR was found in 37 (16.6 %) of all patients with no difference between women and men. Table 1 lists the characteristics of the patients. Patients with positive TaVR were older (*p* < 0.001), more often had a history of MI (*p* = 0.008), had higher prevalence of diabetes mellitus (*p* = 0.012), and were more likely to be on beta-blocker therapy (*p* = 0.019). With regard to echocardiographic parameters, patients with positive TaVR had higher LVMI (*p* < 0.001) and lower EF (*p* < 0.001) in comparison to patients with negative TaVR. With regard to ECG parameters, patients with positive TaVR had increased width of QRS complex and QT interval (*p* < 0.001 and *p* = 0.007, respectively), higher prevalence of ST-segment elevation in lead aVR (*p* < 0.001), and higher prevalence of ST depression (*p* < 0.001) as well as negative T in leads II and V6 (*p* = 0.002 and *p* < 0.001, respectively) in comparison to patients with negative TaVR. Additionally, patients with positive TaVR had higher calcium × phosphate scores (*p* = 0.007) and higher TnT levels (*p* < 0.001). More than 70 % of patients with ST elevation in aVR had concomitant positive TaVR.

**Table 1:** Baseline characteristics

Variable	All patients	TaVR		*p*
	(*n* = 223)	Negative (*n* = 186)	Positive (*n* = 37)	
Men/women (*n*)	0.94	0.94	0.95	0.843
Age (years)	69.57 ± 5.96	68.31 ± 6.53	75.78 ± 6.25	<0.001
HD duration (months)	67.67 ± 6.96	67.29 ± 7.14	69.57 ± 5.76	0.551
MI (%)	26.0	22.0	45.9	0.008
Smoking (%)	18.4	18.8	16.2	0.411
Beta-blockers (%)	71.3	68.8	83.8	0.019
ACE/ARB	75.3	76.3	70.3	0.212
Statins	36.3	35.5	40.5	0.248
Hypertension (%)	75.8	74.7	81.1	0.119
Diabetes mellitus (%)	37.2	34.4	61.4	0.012
Body mass index (*n*)	25.97 ± 2,44	25.99 ± 2.69	25.34 ± 2.24	0.781
Heart rate (b.p.m.)	80.3 ± 4.45	79.4 ± 4.42	84.8 ± 4.66	<0.001
QRS duration (ms)	107.9 ± 19.47	105.4 ± 18.56	120.59 ± 17.87	<0.001
QTc Bazett (ms)	432.6 ± 33.5	430.9 ± 35.8	441.2 ± 29.5	0.007
Negative T in lead II (%)	13.4	10.2	29.7	0.002
Negative T in lead V6 (%)	18.8	13.4	45.9	<0.001
ST depression (%)	25.6	19.4	56.8	<0.001
ST elevation in lead aVR (%)	18.4	7.5	73.0	<0.001
LVMI (g/m^2^)	146.5 ± 45.18	143.7 ± 46.22	159.8 ± 43.11	<0.001
EF (%)	54.91 ± 7.21	55.82 ± 7.28	50.29 ± 8.42	<0.001
Haemoglobin (g/dL)	11.25 ± 1.46	11.21 ± 1.39	11.52 ± 1.79	0.498
Sodium (mmol/L)	138.2 ± 2.5	138.2 ± 2.6	138.0 ± 2.1	0.627
Potassium (mmol/L)	5.83 ± 0.81	5.84 ± 0.79	5.76 ± 0.92	0.654
Calcium × phosphate product (mg^2^/dL^2^)	48.96 ± 10.32	47.28 ± 11.56	57.40 ± 9.79	0.007
Total cholesterol (mg/dL)	186.3 ± 41.05	186.8 ± 41.56	183.1 ± 43.8	0.733
LDL cholesterol (mg/dL)	112.3 ± 32.03	112.5 ± 33.23	111.0 ± 37.68	0.427
HDL cholesterol (mg/dL)	40.41 ± 16.19	40.38 ± 16.08	40.6 ± 17.62	0.426
Triglycerides (mg/dL)	168.8 ± 71.69	168.4 ± 69.02	170.1 ± 70.1	0.415
Albumin (g/dL)	3.81 ± 0.37	3.83 ± 0.39	3.76 ± 0.40	0.252
CRP (mg/dL)	7.38 (0.32–19.9)	7.09 (0.32–13.9)	9.93 (7.32–19.9)	0.191
PTH (pg/mL)	379 (18.0–1736)	361 (18.0–1736)	401 (67.7–1350)	0.255
Troponin T (μg/L)	0.057 (0.00–0.813)	0.051 (0.00–0.533)	0.152 (0.00–0.813)	<0.001
Kt/V (*n*)	1.442 ± 0.275	1.442 ± 0.281	1.446 ± 0.269	0.402

*MI* myocardial infarction, *ACE/ARB* angiotensin-converting enzyme inhibitors/angiotensin receptor blockers, *EF* ejection fraction, *QTc* QT interval corrected for heart rate using the Bazett formula (QTc = QT/√RR), *LVMI* left ventricular mass index, *PTH* partahormon, *Kt/V* number used to quantify hemodialysis dialysis treatment adequacy

Over the mean follow-up period of 25.43 ± 3.56 months (range 1–36 months), there were 79 all-cause deaths, 54 CV deaths including 25 SCD. Five patients were transplanted. The incidence of CV death was as follows: in negative TaVR group 18.8 % and in positive TaVR group 45.9 % (*p* < 0.001). The incidence of SCD was as follows: in negative TaVR group 8.1 % and in positive TaVR group 24.3 % (*p* < 0.001). All transplanted patients came from the negative TaVR group.

**Fig. 2:**
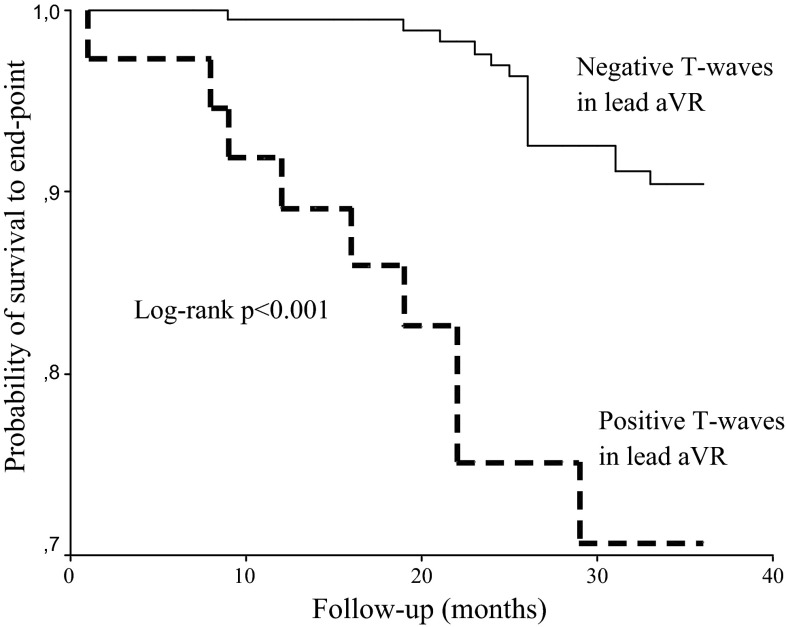
Kaplan–Meier survival plots for cardiovascular mortality in hemodialysis patients stratified by positive and negative T-wave in lead aVR

A Kaplan–Meier analysis showed that the cumulative incidence of CV mortality was significantly higher in patients with positive compared to negative TaVR (log-rank, *p* < 0.001; Fig. 2). To control for possible confounders, multivariate analysis was performed using a model consisting of univariate predictors of cardiac mortality. The results of the univariate and multivariate Cox proportional hazard regression analyses are shown in Table 2. Univariate regression analysis showed that age, history of MI, EF, TnT level, heart rate, QRS duration, ST-segment elevation in lead aVR, and positive TaVR were univariate predictors of CV mortality. A multivariate analysis selected age [hazard ratio (HR) 1.71, *p* < 0.001], heart rate [HR 1.42, *p* = 0.016], and positive TaVR [HR 2.21, *p* = 0.001] as independent predictors of CV mortality.

**Table 2:** Uni- and multivariate predictors of cardiac mortality

Parameter	Univariate HR (95 % CI)	*p*	Multivariate HR (95 % CI)	*p*
Age	1.25 (1.02–1.1.41)	<0.001	1.71 (1.51–1.90)	<0.001
Male gender	0.98 (0.78–1.21)	0.734		
Diabetes mellitus	1.38 (0.85-1.96)	0.133		
History of MI	1.42 (0.91–2.11)	0.012		
LVMI	1.28 (0.69–2.35)	0.279		
EF	2.35 (1.25–2.77)	0.008	1.89 (0.85–3.61)	0.353
Troponin T	2.14 (1.32–3.07)	0.003	1.45 (0.93–2.72)	0.071
Calcium × phosphate product	1.71 (1.07–2.34)	0.228		
Beta-blockers	1.09 (0.78–2.67)	0.677		
Heart rate (10 b.p.m. increase)	1.58 (1.05–2.57)	0.006	1.42 (1.03–1.44)	0.016
QRS (10 ms increase)	1.29 (1.17–1.49)	0.010		
Corrected QT interval	1.18 (0.82–1.84)	0.358		
Negative T in lead II	1.26 (0.80–2.23)	0.125		
Negative T in lead V6	2.11 (1.27–2.97)	0.009		
ST depression in lead V5	1.79 (0.71–3.47)	0.296		
ST elevation in lead aVR	1.73 (1.25–1.89)	0.004	1.69 (0.97–2.46)	0.202
Positive TaVR	3.03 (2.56–4.21)	<0.001	2.21 (1.76–2.79)	0.001

In the multivariate analyses, parameters with a *p* ≤ 0.01 were entered

*HR* hazard ratio, *CI* confidence interval, *MI* myocardial infarction, *LVMI* left ventricular mass index, *EF* ejection fraction

**Fig. 3:**
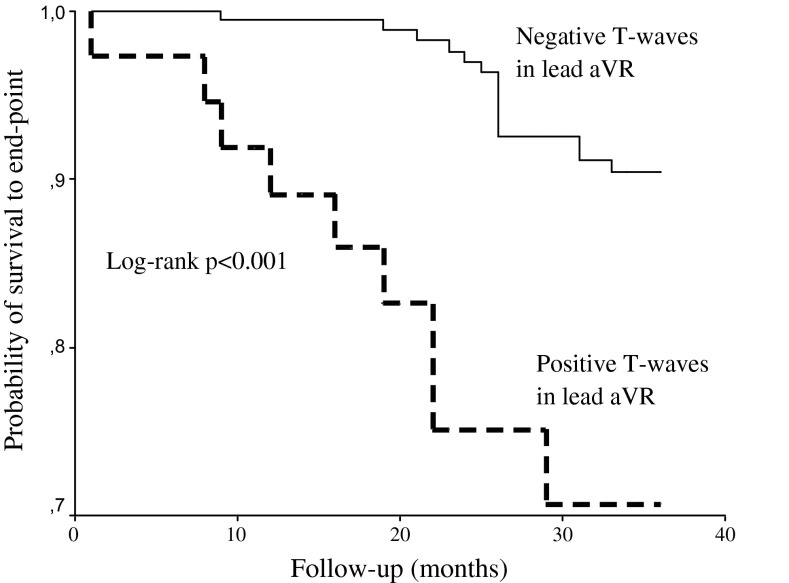
Kaplan–Meier survival plots for sudden cardiac death in hemodialysis patients stratified by positive and negative T-wave in lead aVR

Kaplan–Meier sub analyses were performed to assess the prognostic value of TaVR with regard to SCD. Figure 3 shows that the cumulative incidence of SCD was significantly higher in patients with negative compared to positive TaVR (log-rank, *p* < 0.001). To control for possible confounders, multivariate analysis was again performed using a model consisting of univariate predictors of SCD. The results of the univariate and multivariate Cox proportional hazard regression analyses are shown in Table 3. Univariate regression analysis showed that age, history of MI, LVMI, TnT, and positive TaVR were univariate predictors of SCD. After controlling for univariate predictors of SCD age [HR 1.88, *p* < 0.001], and positive TaVR [HR 1.53, *p* = 0.014] remained independent predictors of SCD.

**Table 3:** Uni- and multivariate predictors of sudden cardiac death

Parameter	Univariate HR (95 % CI)	*p*	Multivariate HR (95 % CI)	*p*
Age	1.15 (1.07–1.30)	<0.001	1.88 (1.45–2.03)	<0.001
Male gender	1.08 (0.69–1.47)	0.804		
Diabetes mellitus	0.88 (0.25–2.37)	0.533		
History of MI	1.84 (1.13–2.98)	0.035		
LVMI	1.44 (1.14–2.21)	0.018		
Ejection fraction	2.14 (1.19–2.68)	0.009	2.35 (0.97–3.87)	0.294
Troponin T	1.64 (1.02–2.97)	0.014		
Calcium x phosphate product	1.01 (0.37–2.17)	0.245		
Beta-blockers	0.95 (0.33–2.47)	0.714		
Heart rate (10 b.p.m. increase)	1.33 (0.98–2.47)	0.203		
QRS (10 ms increase)	1.19 (0.87–1.66)	0.253		
Corrected QT interval	1.48 (0.91–2.74)	0.309		
Negative T in lead II	1.67 (0.76–2.53)	0.311		
Negative T in lead V6	1.43 (1.86–2.57)	0.023		
ST depression in lead V5	1.33 (0.61–3.37)	0.325		
ST elevation in lead aVR	2.23 (0.87–3.49)	0.076		
Positive TaVR	2.54 (2.12–3.59)	0.001	1.53 (1.12–2.74)	0.014

In the multivariate analyses, parameters with a *p* ≤ 0.01 were entered

*HR* hazard ratio, *CI* confidence interval, *MI* myocardial infarction, *LVMI* left ventricular mass index, *EF* ejection fraction

## Discussion

This study showed that TaVR analysis provides prognostic information for HD patients beyond what is available from other known traditional risk factors. The presence of positive TaVR remained an independent and powerful predictor of CV mortality as well as SCD even after adjustment for established CV risk factors. Positive TaVR was superior to any other ECG findings in the identification of patients most at risk for cardiac events. The Kaplan–Meier curves began to separate early and then continued to stay separate until the end of follow-up for both CV mortality and SCD.

Lead aVR often yields information from the right upper side of the heart that is not as readily available from other leads [21]. When repolarization of injured myocardial cells is delayed compared to normal cells, the direction of the T-wave vector changes towards the injured myocardial region [22]. Thus, positive T-waves in lead aVR might represent the presence of ischemically injured myocardium in the apical, inferior, and lower lateral regions of the heart. These areas of the heart are supplied with blood from left anterior descending (LAD), right coronary, or left circumflex coronary artery. Therefore, disease of these arteries or lesions located more proximally in left main coronary artery (LMCA) would be expected to positively invert a normally negative TaVR [21, 23].

The prevalence of positive TaVR in our study (16.6 %) was higher than the general population (2.2 %) [13], and comparable to the prevalence of positive TaVR in patients with heart failure (17.5 %) [14] and prior myocardial infarction (16.2 %) [17]. Previous studies have found that positive TaVR is associated with LMCA disease, multivessel disease, and proximal LAD occlusion [14–16, 21, 23]. It is well established that ESRD is associated with premature and advanced atherosclerosis. Significant coronary artery stenosis affects 30–60 % of asymptomatic HD patients. Moreover, multivessel disease is observed in most cases and LMCA disease is also very common in HD patients [24, 25].

However, in our study TaVR was associated with CV events independent of TnT which is good surrogate marker of myocardial injury. It may be due to the fact that TnT elevation in HD patients may result from reduced renal clearance, direct injury to myocardial cells (toxins, hypoxia, stretching), reversible myocardial ischaemic release in the absence of myocardial necrosis and, from LVH. Elevated TnT levels have been observed in 30–75 % of HD patients in the absence of an acute myocardial injury [26]. Similarly, TaVR influenced CV events independent of EF. Our results are in agreement with some previous studies indicating that the predictive accuracy of EF for CV events is limited [27].

In our study more than 70 % of patients with ST-segment elevation in lead aVR also had positive TaVR. Our study confirms previous observations of Bedheka et al. [13] that ST-segment elevation in lead aVR is predictive of cardiac mortality only when not accounting for positive TaVR. The effect of ST-segment elevation in lead aVR predicting cardiac events can be attributed in part to the presence of positive TaVR.

The prognostic utility of upright TaVR for predicting CV mortality in HD patients is in concordance with previous studies that have documented that positive TaVR is a powerful marker for estimating risk of CV death both in the general population [12, 13] as well as in some clinical settings such as heart failure, acute coronary syndromes, or myocardial infarction [14, 15, 28, 29].

To our knowledge, this is the first study that has demonstrated the prognostic utility of upright TaVR for predicting SCD. Given the ubiquity, inexpensiveness and convenience of ECG recordings and the fact that SCD is the single largest cause of death in HD patients [1–3, 5], the results of TaVR might be readily utilized for SCD risk estimation in HD patients. It currently remains unclear whether upright TaVR is merely a marker of poor prognosis that reflects the high prevalence of the most severe forms of coronary artery disease, contributing to CV mortality, including SCD, or whether it potentially identifies more distinct electrophysiological mechanisms that underlie increased mortality and SCD. Such mechanistic questions require further investigation.

## Study limitations

The present study has some important limitations. First, the numbers of patients in the study was relatively small. Nevertheless, the population was large enough to demonstrate a predictive value for a positive TaVR. Second, the exclusion of patients with some clinical conditions potentially associated with a higher mortality, such as atrial fibrillation, may also add bias to the results. Third, it is possible that serial rather than single measurements of TaVR may have changed the results, making TaVR either more or less useful in predicting clinical events in HD patients.

## Conclusions

Positive TaVR is associated with increased CV mortality as well as SCD in HD patients. This simple ECG parameter provides additional information beyond what is available with other known traditional risk factors and allows the identification of patients most at risk of CV events. HD patients with a positive TaVR, should be considered for additional diagnostic and preventive measures.
